# Linking Podcasts With Social Media to Promote Community Health and Medical Research: Feasibility Study

**DOI:** 10.2196/10025

**Published:** 2018-10-24

**Authors:** Joyce Balls-Berry, Pamela Sinicrope, Miguel Valdez Soto, Tabetha Brockman, Martha Bock, Christi Patten

**Affiliations:** 1 Department of Epidemiology Mayo Clinic Rochester, MN United States; 2 Center for Clinical and Translational Science Mayo Clinic Rochester, MN United States; 3 Behavioral Health Research Program Mayo Clinic Rochester, MN United States; 4 Department of Psychiatry and Psychology Mayo Clinic Rochester, MN United States

**Keywords:** biomedical research, community health, community and patient engagement in research, podcast, social media

## Abstract

**Background:**

Linking podcasts with social media is a strategy to promote and disseminate health and health research information to the community without constraints of time, weather, and geography.

**Objective:**

To describe the process of creating a podcast library and promoting it on social media as a strategy for disseminating health and biomedical research topics to the community.

**Methods:**

We used a community and patient engagement in research approach for developing a process to use podcasts for dissemination of health and health research information. We have reported the aspects of audience reach, impressions, and engagement on social media through the number of downloads, shares, and reactions posted on SoundCloud, Twitter, and Facebook, among others.

**Results:**

In collaboration with our local community partner, we produced 45 podcasts focused on topics selected from a community health needs assessment with input from health researchers. Episodes lasted about 22 minutes and presented health-related projects, community events, and community resources, with most featured guests from Olmsted County (24/45, 53%). Health research was the most frequently discussed topic. Between February 2016 and June 2017, episodes were played 1843 times on SoundCloud and reached 1702 users on our Facebook page.

**Conclusions:**

This study demonstrated the process and feasibility of creating a content library of podcasts for disseminating health- and research-related information. Further examination is needed to determine the best methods to develop a sustainable social media plan that will further enhance dissemination (audience reach), knowledge acquisition, and communication of health topics.

## Introduction

Social media presents a powerful tool for reaching, engaging, and connecting individuals for public health and health promotion [1]. Social network platforms have a large reach at relatively low cost, representing a distinct advantage over face-to-face approaches [2]. Social media is used for promoting health literacy through education and dissemination of information to the lay public [3] to encourage behavior changes [4] (eg, smoking cessation, diabetes prevention, exercise) and promote research participation [5], among others. In addition, social media has been utilized among health professionals for continuing education [6] and dissemination of clinical practice changes [7]. Social media (ie, Web 2.0 technologies) can increase the depth of engagement and connection with extensive reach to underserved, diverse populations [8,9] and with evidence-based content.

Integrating technology, such as podcasts into traditional health care communication or dissemination models, is an effective and practical strategy for not only delivering quality health-related information to the public, such as clinical practice changes, education, and health research [10,11], but also for creating opportunities for engagement with the content [12,13]. Podcasts are downloadable, digital, episodic audio recordings streamed through a Web-based platform that can be easily accessed using portable media players and then shared on multiple social media platforms such as Facebook, Twitter, or blogs. Over time, the number of people listening to podcasts has increased, especially among those interested in health care, research, and education [11-14]. Between 2013 and 2016, 21% of Americans aged ≥12 years have reported listening to a podcast in the previous month, a steady 36% increase from 2008 to the present that coincides with use and access to smartphones, tablets, and other mobile devices [14,15]. This strategy fits well into the principles of community and patient engagement in research (CPER) [16] that aim to establish sustainable ways to build trust, respect culture, and create clear expectations of outcomes to increase wellness. Traditional CPER approaches have involved intensive face-to-face communication formats to promote communication between researchers, community members, and other stakeholders [17-19]; yet these approaches limit audience reach and can restrict participation due to constraints of time, geography, and weather. Podcasts, on the other hand, can accomplish similar aims, but are subject to none of these limitations; their content can be created based upon the interests and listening patterns of their audience. The process of creating a community topic driven podcast afforded us the opportunity to design a dissemination tool to raise awareness about health and biomedical research. After creating our podcasts, we realized that our experiences might benefit others with community-academic partnerships with similar challenges. Moreover, we realized that our approach was a novel way to share health- and community-related information using social media platforms to stimulate communication and maximize audience reach.

## Methods

### Developing Podcasts

The idea for developing podcasts arose from a discussion during a community advisory board meeting about the need to transfer the content of a Science Café to the Web-based realm. Science Cafés are in-person, casual events that encourage two-way conversations about health- or science-related topics between scientists, health practitioners, and the lay public to enhance health literacy, trust, and colearning. A series of these events had been recently conducted in the community, and they were termed as “garden cafés,” as many had taken place in community gardens. While successful in stimulating conversation and idea exchange, the reach of these cafés was limited due to the barriers of geography, weather, and time. Therefore, the decision was made to create a series of health-related podcasts based upon a recent community health needs assessment that would provide an extensive library of topics to share via social media. Therefore, our team decided to incorporate another social media platform to disseminate community health and medical research information. The Mayo Clinic Center for Clinical and Translational Science’s Community Engagement Program (CEP) had a strong social media presence. The program had a WordPress blog, Twitter page, and Facebook page [8]. One area that we decided to explore was the creation of a community-academic podcast. Like the cafés, we planned to use a similar process to determine topics and considered this part of what we called our Social Media Community Garden Café approach.

The development of meaningful podcast content was supported by CEP and Smartride Network. Our partner, Smartride Network, is a collaborative, community-based organization that hosts a variety of cultural, art, health, and biomedical research programs. To select topics for podcasts, we referred the Olmsted County Community Health Needs Assessment (CHNA) [20]. The CHNA not only recommended health topics, but also stated that new social media approaches should be developed for disseminating information within the growing Olmsted County community. This article reports the process that stakeholders used to develop podcast topics and promote the podcasts themselves on social media, as well as the basic analytics of audience reach, impressions, and engagement as reported through the number of downloads, shares, and reactions posted on SoundCloud, Twitter, and Facebook.

The podcast team decided to focus the first few episodes on community-identified health and wellness needs. The systematic community-engaged approach involved Olmsted County Public Health Services, Olmsted Medical Center, Mayo Clinic, and several community service organizations jointly collecting data from residents on their health needs [20]. The organizers of the CHNA held a number of community events as well as other activities with local community members and community organizations to prioritize the most pressing health topics locally. The top 5 topics included: injury prevention, immunizations, overweight or obesity, mental health, and financial stress. After the initial episodes, the podcast team expanded the content list to topics related to cultural events, health and wellness, and international health. The change in content selection also expanded the pool of guest speakers for the podcast. Local media and internal communications for events like academic seminars provided other sources of inspiration for the podcast.

Guests were invited in-person by the host or other team members to share their stories. We determined the credibility of guest speakers by exploring local content expertise. For national and international speakers, we connected with the organizers of a number of speaker series at our academic medical center (eg, Grand Rounds and other medical seminars) to ask visiting faculty to create a podcast during their visit. Guests shared their stories related to community priorities and biomedical research.

The episode format consisted of the following:

- Welcome and introduction by the host and guest
- Guest describes the personal journey that led to current work
- Guest shares current projects, findings, resources, and engagement opportunities for listeners
- Guest creates the titles of the episodes with the host to enhance promotion in their networks
- Host concludes each episode with a call-to-action to follow our social media platforms and for listeners to share the podcasts with their networks

We used a CPER research approach to determine sponsorship for podcast recording equipment and to prioritize episode creation. This approach involved working directly with the CEP, local community partners, and health care providers. The podcast design process focused on audience and delivery. We recognized that the podcast alone does not foster communication and information dissemination like other forms of social media. Therefore, it was linked to other interactive social media tools such as Twitter and Facebook, where listeners could comment and post reactions.

### Audience

Our primary target audience for the podcasts was residents of Olmsted County, MN, with an interest in learning about (1) health and wellness; (2) community events; (3) opportunities for civic engagement; and (4) biomedical research. The podcast featured guests from local agencies, nonprofits, the health department, community members, and academic researchers (local, national, and international) [9].

### Podcast Hosting

SoundCloud was selected as the main hosting platform for the podcast [21]). This platform allows members to share digital audio content in the form of a podcast. It also allows broadcasters to create albums and playlists for their recorded and posted content. Our podcast was shared on iTunes through a plug-in of the Rich Site Summary feed. Podcast episodes are sharable over other forms of social media.

### Episode Design

#### Podcast Promotion

An open, searchable Facebook page, called Community Board, was created for consistency in branding the community podcast initiative. The episodes were listed on the CEP WordPress blog and Twitter account. A Rich Site Summary feed was used to ensure that the podcast was accessible on mobile devices and computers [11,22]. We shared information about new episodes via email and asked community members, guests, and others interested in the topics to share the podcast link with colleagues and stakeholders. The podcast stakeholders were also members of the Olmsted County Community Needs Assessment Team.

#### Assessing Podcast Reach

We assessed podcast reach using available social media analytics from SoundCloud and our CEP Facebook and Twitter pages. The SoundCloud analytics allowed us to capture information on social media engagement (ie, likes, reposts, and comments). Other analytics provided information on (1) episode length in minutes and seconds, (2) number of times the podcast was played, and (3) geographic location of listeners [23]. Episodes were grouped by three focus areas (ie, biomedical research, art and cultural events, and local resources) and by guest speakers’ geographic location. SPSS (IBM SPSS Statistics for Windows, Armonk, NY, USA) was used to calculate descriptive information including frequencies and measures of central tendency.

## Results

The Community Board Podcast produced 45 episodes with 1843 cumulative plays, 728 of which occurred between February 2016 and June 2017 (Table 1). Our Facebook page has 72 subscribed followers and has reached 1702 listeners. Just over half of the episodes featured local speaker(s) from Olmsted County, MN (24/45, 53%), with the other half of speakers from organizations in the state of Minnesota (3/45, 7%), the United States (14/45, 3%), and outside the United States (4/45, 9%). Biomedical research topics garnered the greatest number of listeners (1058/1843, 57.40%), followed by community resource awareness (417/1843, 22.63%) and information on art and cultural events (368/1843, 19.97%).

In the first 6 months of the podcast, 21 episodes were produced (Table 2), most of which aired in April (7/21, 33%). However, the greatest number of total downloads occurred during the month of March (163/728, 22.4%). During the first 6 months of each airing, the episode that was played the most was The Great Lakes Inter-Tribal Council (87/725, 12.0%). This topic was selected in collaboration with a community partner and research team focused on Native American health. Overall, there were 42 “likes” (indicating a positive reaction to the podcast), with most of the “likes” occurring in May (17/42, 40%). Nearly two-thirds of the listeners listened to the podcast via SoundCloud platforms (498/722, 68.98%).

From February 2016 to June 2017, the top 5 episodes played were (1) Women’s Health-Uterine Fibroids (n=119), (2) The Great Lakes Inter-Tribal Council, Incorporated (n=104), (3) Wellconnect SE MN (n=80), (4) Black History Month Meet the Researcher–Camara Phyllis Jones, MD (n=72), and (5) Music is Medicine to the People (n=66). The average episode length was 22 minutes and 19 seconds (SD 12 minutes and 25 seconds). The podcast received 1846 cumulative plays between February 2016 and June 2017. There were 1653 listeners in the United States, with 590 in Olmsted County, MN, and 193 were from other countries. The top played episodes featured guests with a strong social media presence. After publishing the episodes, we linked them directly to our WordPress blog, Facebook, and Twitter accounts, allowing the guests to share the posts with their stakeholders.

**Table 1:** Podcast episodes since February 2016.

Episode titles	Geographic focus	Episode theme	Publication date	Length in minutes	Total plays
Introduction to Community Board	Local	Resource	February 2016	8:07	44
KnuFunk Band	Local	Art & Culture	February 2016	14:47	43
Three Rivers Community Action	Local	Resources	February 2016	11:50	22
Black History Month Meet the Researcher – Camara Phyllis Jones, MD	National	Research	February 2016	14:22	72
Artist Bobby Marines’ New Work	Local	Art & Culture	March 2016	18:00	52
Wellconnect SE MN	State	Research	March 2016	16:29	80
Women’s History Month	Local	Art & Culture	March 2016	8:47	36
stART-up Fund Program	Local	Art & Culture	April 2016	10:24	21
Women’s Health-Uterine Fibroids	National	Research	April 2016	22:10	119
RNeighbors #RochMN	Local	Resource	April 2016	16:53	32
Dr. Paul Spaicer Learning with Natives Communities	National	Research	April 2016	22:53	68
Black Hair Politics of Beauty	Local	Art & Culture	April 2016	12:14	52
Airbnb and Research	International	Research	April 2016	18:33	26
Art on the Ave	Local	Art & Culture	April 2016	16:19	25
Learn about Uterine Fibroids and Underwater Hockey	National	Research	May 2016	21:01	66
The Commission #RochMN	Local	Resource	May 2016	28:12	31
#Prince, Blues, and BBQ	Local	Resource	May 2016	22:04	47
Mission 21 Sex Trafficking	Local	Resource	May 2016	53:18	35
The Great Lakes Inter-Tribal Council, Inc.	National	Research	June 2016	32:30	104
Affordable Housing-Volunteer Opportunities	Local	Resource	June 2016	26:45	41
Juneteenth Commemorating the Ending of Slavery in US	Local	Art & Culture	June 2016	10:39	31
Community Prioritization Session #CHNA	Local	Research	July 2016	11:35	32
NAMI Mental Health Resources	Local	Resource	September 2016	30:02	30
Day of the Dead Poet Slam	Local	Art & Culture	October 2016	20:50	30
Your Local Farmers Market	Local	Research	October 2016	24:09	40
BMG Basement Music Group	Local	Art & Culture	November 2016	18:06	43
MondayCampaings.org	National	Research	November 2016	8:59	34
Clinical-Scholars.org	National	Research	November 2016	19:35	34
Community-Campus Partnerships for Health (CCPH)	International	Research	November 2016	13:28	48
Men’s Health Caucus 1	National	Research	November 2016	8:12	43
Men’s Health Caucus 2	National	Research	Novembers 2016	9:50	24
Men’s Health Caucus 3	National	Research	December 2016	18:30	30
Improving Health Globally by Studying Health Locally	National	Research	December	33:27	51
Youth and Men’s Health Caucus 4	National	Research	December 2016	7:05	29
Working Together to Strengthen Our Future	Local	Resource	December 2016	22:38	44
Music is Medicine to the People	National	Research	January 2017	52:10	66
The Power of Movement Dr. James Levine	National	Research	January 2017	56:59	41
Community Interfaith Dialogue on #islam	Local	Art & Culture	February 2017	44:44	35
Girls on the Run #RochMN	Local	Resource	March 2017	16:44	22
Diversity Council	Local	Resource	April 2017	24:12	32
South Side Community Health Services	State	Research	April 2017	22:28	27
Main Street Project	State	Resource	May 2017	46:02	30
Indigenous Smoking An Australian Perspective	International	Research	May 2017	28:20	19
Health Disparities Study in South Africa	International	Research	May 2017	29:43	5
Bee Keeping Year 1	Local	Resource	June 2017	30:23	7

**Table 2:** Analytics for the first 6 months of community board podcasts.

Analytics		Values n (%)
**Episodes produced during first 6 months (n=21)**		
	February	6 (28.6)
	March	3 (14.3)
	April	7 (33.3)
	May	2 (9.5)
	June	2 (9.5)
	July	1 (4.8)
**Total listeners by month (n=728)**		
	February	103 (14.1)
	March	163 (22.4)
	April	189 (26.0)
	May	116 (15.9)
	June	146 (20.1)
	July	11 (1.5)
**Total likes (n=42)**		
	February	6 (14.3)
	March	5 (11.9)
	April	6 (14.3)
	May	17 (40.5)
	June	8 (19.0)
	July	0 (0)
**Reposts (n=2)**		
	February	0 (0)
	March	0 (0)
	April	0 (0)
	May	1 (50.0)
	June	1 (50.0)
	July	0 (0)
**Platforms for playing podcasts (n=722)**		
	SoundCloud Apps, Embedded, and Mobile	498 (69.0)
	Apple Core Media and iTunes	80 (11.1)
	Rich Site Summary Feed	144 (19.9)

In addition to determining the number of plays, we wanted to see additional social media engagement of the podcast. During the reporting period, we had 70 likes for the podcasts, suggesting that these listeners “liked” or enjoyed that episode. We also had a total of 4 reposts from listeners to other social media sites. The analytics did not allow us to see beyond one repost from our originating site, so it is possible that there were further shares or reposts, but they cannot be tracked. We had 3 listeners comment on episodes and 3 listeners download episodes.

## Discussion

### Principal Findings

The Community Board Podcast produced 45 episodes with most featuring local speakers. Most of the listeners for the podcast were from the United States, with a small portion from other countries. We used existing social media platforms to promote the podcasts and asked community stakeholders to promote the episodes to their stakeholders. The episodes that garnered the greatest number of listeners were those related to biomedical research topics, with women’s health having the most total plays.

Podcasts are an emerging strategy for dissemination of pertinent issues in the community with the potential to raise awareness and increase knowledge of health and wellness as well as biomedical research. We purposefully selected podcasts as a social media platform because they allowed us to create quality content that was sharable within the community without limitations of time, audience reach, geography, or weather with our stakeholders. This platform has proven to be effective with diverse communities. Olmsted County, MN, is the 8^th^largest county in the state, with Hennepin County being the largest with continued growth in the community due to a new public-private economic initiative called Destination Medical Center [24,25]. The growing changes in our community have influenced the need to identify the most effective methods to share information related to health and wellness with community stakeholders. The increased use of mobile devices by patients, patient advocates, and community members allows new ways for academic medical centers to connect with stakeholders and increase knowledge about wellness and biomedical research. This use of a podcast in our local community facilitated a new approach for promoting communication and awareness of biomedical research and cultural events and information about local community resources (14). Additionally, the Community Board Podcast’s reach extended beyond the local community, with downloads recorded throughout the United States and beyond. This unexpected reach in a global community is notable because the topics reflected not only the interests of the local community but also the interests of a larger global audience.

### Strengths and Limitations

Using health priorities identified by community members through the CHNA as the basis for the initial podcasts proved to be a strength. These topics led to more direct conversations with stakeholders (ie, community members, patients, service providers, and biomedical researchers) on potential topics of interest. Another strength was the ease in linking podcasts to other social media platforms, which allowed a larger audience to experience, interact with, and discuss the podcasts in their own social networks. Unfortunately, we were unable to fully track the depth of reach of our podcasts with the available analytics or to capture an increase in knowledge since we did not include an evaluation measure for knowledge gain. Future studies should use more in-depth analytic programs for tracking audience reach and interaction. Future studies could employ experimental designs to assess knowledge gain, audience reach, and longer-term consequences on health and wellness, clinical practice, and biomedical research.

One significant strength and key feature of the podcast is that it relates directly to recommendations from Dr Christopher Austin, Director of National Center for Advancing Translational Sciences at the National Institutes of Health (NIH), who advised us in an interview that researchers, “must talk in language the public can understand” [26]. Podcasts are an emerging platform that biomedical research programs can use to directly connect with the community in places where they will hear it and in language they will understand. Moreover, actively engaging community members and researchers in continued bidirectional dialogues helps reduce the time needed to raise the general population’s awareness and understanding of research findings and how they address community-related needs [27].

While our results show that developing and promoting health- and research-related podcasts is feasible, more research is needed to determine the best ways to develop and promote these educational sessions to maximize audience reach (ie, number of downloads, shares, reactions, and reposts). An understanding of audience reach will stimulate colearning, thus increasing the knowledge of both community members and health care practitioners or researchers. For instance, podcasts are a platform often used in Free Open Access Medical education (FOAM). The FOAM movement has altered the way health care practitioners interact with each other and serves as a supplement to traditional pedagogical methods to increase the knowledge of medical learners [28,29]. Moreover, the use of social media platforms like podcasts promotes the translation of evidence-based medicine to the medical community to increase knowledge [30]. Lessons learned from the use of podcasts in the FOAM movement are translational to increase research literacy and overall knowledge of health in the community.

### Future Work

Future directions for the podcast include developing and evaluating novel ways to better track active engagement and knowledge increase of listeners. The evaluation process should include the application of Kirkpatrick’s model level 1 and 2 to determine listeners’ reactions and knowledge gain from listening to the podcast [31]. We may also consider including moderated postings to our existing social media pages that will encourage direct communication about health topics and health research to aid in the evaluation process. This process may also include the use of Facebook Live or other video blogging tools to facilitate real-time communication with listeners. This process could increase bidirectional communication, engagement, and knowledge with health care experts and the community. Moreover, the addition of another theoretical framework, such as Reach, Effectiveness, Adoption, Implementation, and Maintenance, may provide more consistency and foster a process to determine individual and institutional impact [32,33]. Finally, we found that episodes that focused on community-identified topics and promoted by stakeholders were the most popular. Therefore, more effort is needed to devise a standardized community promotion plan to further extend our audience reach to address other health and health research topics. For example, for future work, we plan to develop a toolkit to guide community organizations on the best methods and ways to promote podcasts developed in partnership.

### Conclusion

In conclusion, our study suggests that podcasts linked with other social media platforms are a feasible strategy for sharing and stimulating communication about health and biomedical information to a wide audience without barriers of time, geography, and weather. These preliminary results will inform the development of a large-scale trial of Social Media Garden Cafés (podcasts linked with social media) to educate and empower communities, health providers, and biomedical researchers for improving health care research and delivery.

## Abbreviations

CEP: Community Engagement Program
CHNA: Community Health Needs Assessment
CPER: community and patient engagement in research
FOAM: Free Open Access Medical
NIH: National Institutes of Health
